# The Effect of Incorporating Industrials Wastewater on Durability and Long-Term Strength of Concrete

**DOI:** 10.3390/ma14154088

**Published:** 2021-07-22

**Authors:** Ehsan Nasseralshariati, Danial Mohammadzadeh, Nader Karballaeezadeh, Amir Mosavi, Uwe Reuter, Murat Saatcioglu

**Affiliations:** 1 Department of Civil Engineering, University of Ottawa, Ottawa, ON K1N 6N5, Canada; Enase063@uottawa.ca (E.N.); Murat.Saatcioglu@uOttawa.ca (M.S.); 2 Department of Civil Engineering, Ferdowsi University of Mashhad, Mashhad 9177948974, Iran; Danial.mohammadzadehshadmehri@mail.um.ac.ir; 3 Department of Civil Engineering, Mashhad Branch, Islamic Azad University, Mashhad 9187147578, Iran; 4 Department of Civil Engineering, Faculty of Montazeri, Khorasan Razavi Branch, Technical and Vocational University (TVU), Mashhad 9176994594, Iran; N.karballaeezadeh@shahroodut.ac.ir; 5 Faculty of Civil Engineering, Shahrood University of Technology, Shahrood 3619995161, Iran; 6 Faculty of Civil Engineering, Technische Universität Dresden, 01069 Dresden, Germany; uwe.reuter@tu-dresden.de; 7 John von Neumann Faculty of Informatics, Obuda University, 1034 Budapest, Hungary

**Keywords:** sustainable concrete, wastewater, industrial waste management, sustainable development, sustainable construction materials, circular economy, recycling, materials design, construction materials, materials properties

## Abstract

Concrete, as one of the essential construction materials, is responsible for a vast amount of emissions. Using recycled materials and gray water can considerably contribute to the sustainability aspect of concrete production. Thus, finding a proper replacement for fresh water in the production of concrete is significant. The usage of industrial wastewater instead of water in concrete is considered in this paper. In this study, 450 concrete samples are produced with different amounts of wastewater. The mechanical parameters, such as slump, compressive strength, water absorption, tensile strength, electrical resistivity, rapid freezing, half-cell potential and appearance, are investigated, and a specific concentration and impurities of wastewater that cause a 10% compressive strength reduction were found. The results showed that the usage of industrial wastewater does not significantly change the main characteristics of concrete. Although increasing the concentration of wastewater can decrease the durability and strength features of concrete nonlinearly, the negative effects on durability tests are more conspicuous, as utilizing concentrated wastewaters disrupt the formation of appropriate air voids, pore connectivity and pore-size distribution in the concrete.

## 1. Introduction

In the modern era, concrete is one of the most used materials in the construction industry. In fact, the only other substance that humans use more than concrete is water, which indicates the importance of concrete and the water used for it [1,2,3,4]. Since the first time concrete was utilized as a building material, fresh water was used to cure the cement [5]. The performance of concrete that is made of wastewater has also been investigated; however, further research is essential for examining whether using wastewater is financially feasible and could meet construction standards [6]. There is a research gap in the life cycle assessment, environmental, functional, physical and economic aspects of using wastewater; filling this gap could lead to a revolutionary movement in the construction industry [7]. Bearing in mind the amount of water required for construction projects, if potable water could be substituted with recycled water, it would reduce costs but it would also prevent the wastage of an enormous amount of drinkable water resources [8]. Rivers and fountains that are not contaminated by domestic wastewaters and do not have a salty taste are appropriate for concrete mix designs [9]. Researchers also have indicated that the lake water, which contains less silt, organic materials and impurities, has insignificant adverse effects on concrete features; however, other comprehensive studies are needed on other potential replacements [10]. In industrial and urban areas with limited drinkable resources, and according to fast enhancement in the industry, the demand for water storage is being felt more and more [11]. According to the majority of scientists, the best way to make construction materials is to use the residue of materials, and one of the most prominent construction materials is concrete, of which approximately 5 million cubic meters is used per year globally [11]. This significant value can be seen as an excellent opportunity to use wastewater in concrete, containing 28% of the water cycle [12]. It is undeniable that one of the most usable basic materials in industrial towns is water, which becomes wastewater after use, and is highly harmful to human health and the environment. Concerning the potable water crisis, especially in the Middle East and Africa, finding other water resources as a suitable replacement rather than drinkable water for producing and curing concrete has drawn significant attention, leading to a search for solutions that not only economize cost and energy but also present novel methods. As a result, burying harmful materials and better productivity are obtainable, and less detrimental influences on the environment are expected. According to the United Nations (UN) world water development report, a series of global actions have been taken over five years, costing over 25 billion dollars in order to have healthy infrastructures for water and wastewater [12]. It is worth mentioning that the amounts of produced industrial wastewater and sludge in the United States of America are 119 billion gallons and 17 million tons per year, respectively. These statistics for Europe are 123 billion gallons and 18.9 million tons, respectively [12]. Therefore, according to the huge volume of industrial wastewater and its harmful impacts on the environment, the current study is urgently required. 

In this research, help is provided to find the level of wastewater refinement to be used in concrete production. This can help to keep the wastewater infrastructures well maintained due to the massive amount of caustic materials in industrial wastewater. It defines what amount of impurity in a sample can cause a less than 10% loss of compressive strength, compared with a control sample; this is a crucial factor because it can help with the approval of the use of different types of wastewater as appropriate replacements for drinkable water. Al-Ghusain et al. [13] reported on primary, secondary and tertiary treated wastewater, which was taken from the local wastewater plant. The water they utilized did not change the slump; however, the setting time was increased by lowering the water quality. They explained that impurities in the water of a concrete mix design impose different effects on setting time and strength, and also create some stains on the concrete’s external surface. Not all impurities harm concrete and some reactions can be neutral or even suitable for concrete. Shekarchi [14] used biologically treated wastewater in concrete mixing and curing. Physical and mechanical tests were performed on mortar and concrete cube specimens. Some durability tests of concrete were also evaluated. When the mixing and curing of concrete was carried out in primary and secondary water, the compressive strength increased up to 17% more than in the concrete mixed and cured in tap water, for up to 180 days. After 180 days, concrete that was mixed and cured in primary treated water showed a small reduction, and when secondary treated water was used for mixing or curing in concrete, compressive strengths were decreased from 9% to 18%. The water absorption of the concrete mixed in tap water and that mixed in treated wastewater was identical. Curing in secondary wastewater increased the water absorption of the specimen. These results showed the feasibility of biologically treated water in the concrete production industry. Asadollahfardi et al. [15] studied using concrete wash water to produce concrete. Their results indicated that concrete wash water is suitable for producing fresh concrete. This research is based on the compressive strength, flexural strength, abrasion resistance, chlorine resistance and carbonation resistance of treated wastewater concrete (10%, 25%, 50% and 100% replacement with tap water) and compares the results with control concrete. This research shows the feasibility of using treated wastewater in concrete to reduce the consumption of fresh water in the concrete industry, as well as solving the problem of disposing of industrial wastewater. Asadollahfardi et al. [16] used treated domestic wastewater instead of drinking water to produce and cure concrete samples. Their results indicated that the compressive strength of the samples made with treated domestic wastewater at the age of 28 days was 93–96% of the compressive strength of the control samples made with drinking water. The use of treated domestic wastewater also did not have much effect (less than a 4% decrease in resistance) on the tensile strength of the concrete samples; however, a final setting time of the cement was delayed by 15 min was observed. Domenico et al. [17] conducted research on the structural behavior of RC beams containing EAF slag as recycled aggregate. The authors stipulated that EAF slag has a remarkably higher specific weight (evaluated macroscopically with the pycnometer test method), which provides roughly the upper limit of the slag. This is in principle, because of the high content of metallic iron, iron and manganese oxides (which have a density higher than 5000 kg/m^3^), which compose the slag. Their results indicated that the existence of steel slags results in more shear potential than the traditional RC beams, crack widths are smaller and the basic ductility is augmented. Bahraman et al. [18] carried out research on the feasibility of using both wash water from a ready mixed concrete plant and synthetic wastewater. According to the visual stability index and slump flow time results, they reported that the utilization of either wash water from the ready-mixed concrete plant or synthetic wastewater impose destructive impacts on the workability of concrete in comparison with the control sample. Likewise, it was indicated that outcomes of the J-ring and column segregation index of individual self-compacting concrete escalated in comparison with the control sample. Besides, although the 28 days’ compressive strength of all specimens, using wash water or synthetic wastewater instead of tap water, was reduced, the concrete that contained synthetic wastewater (1000 mg/L total dissolved solid) had 13.335% more compressive strength. Taherlou et al. [19] studied the practicability of using a variety of percentages of simultaneous municipal solid waste incineration bottom ash and treated industrial wastewater in self-compacting concrete. It was illustrated that the workability of different self-compacting concrete mixtures, including different percentages of municipal solid waste, can reach a satisfactory level within the European guidelines (ASTM C1585) by utilizing the rate of the superplasticizer. In addition, the compressive strength increased more by using solid waste and the treated industrial wastewater compared to self-compacting concrete samples using tap water. The SEM images showed fewer pores and cracks while utilizing the treated industrial wastewater in self-compacting concerts. Ali Raza et al. [20] assessed the mechanical and durability behavior of recycled aggregate concrete made with different kinds of wastewater, including that from domestic sewerage, a fertilizer factory, a textile factory, a sugar factory and a service station. It was observed that, by utilizing the wastewater taken from the textile factory, the compressive strength and split tensile strength were 19% and 16% higher compared to concrete produced with drinkable water. Moreover, the specimen made with domestic sewerage for the mixing had 13.88% improvement, which was the highest water absorption of all utilized wastewaters.

Undeniably, concrete production is the main reason for a considerable amount of energy consumption as well as CO_2_ production. Therefore, it is vital to substitute new promising ways in which components are replaced by other materials, yet rapt attention should be paid to recycled keys [21]. Clearly, there is still a research gap in the functional and economical aspects of using wastewater. Due to development in all industrial sectors, which has flourished and enlarged industrial towns as well as increased the human population, coupled with the demand to hamper expenditure in different government budget sectors, attention should be focused on the reuse of resources if possible. In previous studies, the feasibility of using wastewater, including treated domestic wastewater and concrete wash water, as well as primary, secondary and tertiary treated wastewater, was evaluated. Nevertheless, the precise effect of a variety of industrial wastewater types with different concentrations was not specified, especially concerning its behavior in terms of diluting and concentrating. Another important reason to research industrial wastewater is that the result can be expanded to other kinds of wastewater because it has the highest level of impurity and chemical parameters, so any solution for this type of wastewater could be applied to other weaker wastewaters. Thus, detailed research with different durability and strength test ranges, from primary industrial wastewater, a variety of treated wastewater concentrations and a standard control sample, was required. In brief, a few unique results of this research are as follows: (i) presenting an optimum level of industrial refinement for using it in concrete; (ii) providing a vivid understanding of the linearity or non-linearity behavior of specimens and their performances by diluting or concentrating industrial wastewater; (iii) defining the impurity level of a sample that can cause a 10% compressive strength reduction in comparison with a control sample; (iv) the effect of impurity and industrial parameters on concrete specimens, including ITZ region, pore connectivity, air void parameters, pore structure and size-distribution, air content and so forth.

In the present research, different concentrations of industrial wastewater were used for producing concrete specimens. Subsequently, the durability and strength of concrete specimens within 365 days were assessed and compared with the control specimen, which was produced with drinkable water. Eventually, a statistical analysis is presented to augment the level of strength prediction in concrete based on the obtained results.

## 2. Materials and Methods

### 2.1. Method of Examination

The wastewaters were gathered from Toos industrial town, Mashhad, Iran and within a maximum of three hours, they were analyzed in the laboratory. The analyses were performed on industrial primary wastewater, treated wastewater, diluted treated wastewater, and concentrated treated wastewaters. The control specimen was produced with drinkable water from Mashhad City, which is a standard water. Altogether, 430 specimens were created, pouring concrete ten times and fourteen skilled operators participated in producing them, which took two hours in total. The number of completed tests on specimens were as follows: slump 10 samples, compressive strength 240 samples, electrical resistivity 20 samples, water absorption of thirty minutes 10 samples, mass water absorption 10 samples, capillary water absorption 30 samples, tensile strength 40 samples, rapid freezing and thawing 40 samples and half-cell 30 samples. All of the tables, results, and tests were carried out exclusively for this research, and no archive data were used. The Technical and Vocational University (TVU), Mashhad, Iran, provided the researchers with testing facilities. The used standards are shown in Table 1.

**Figure 1:**
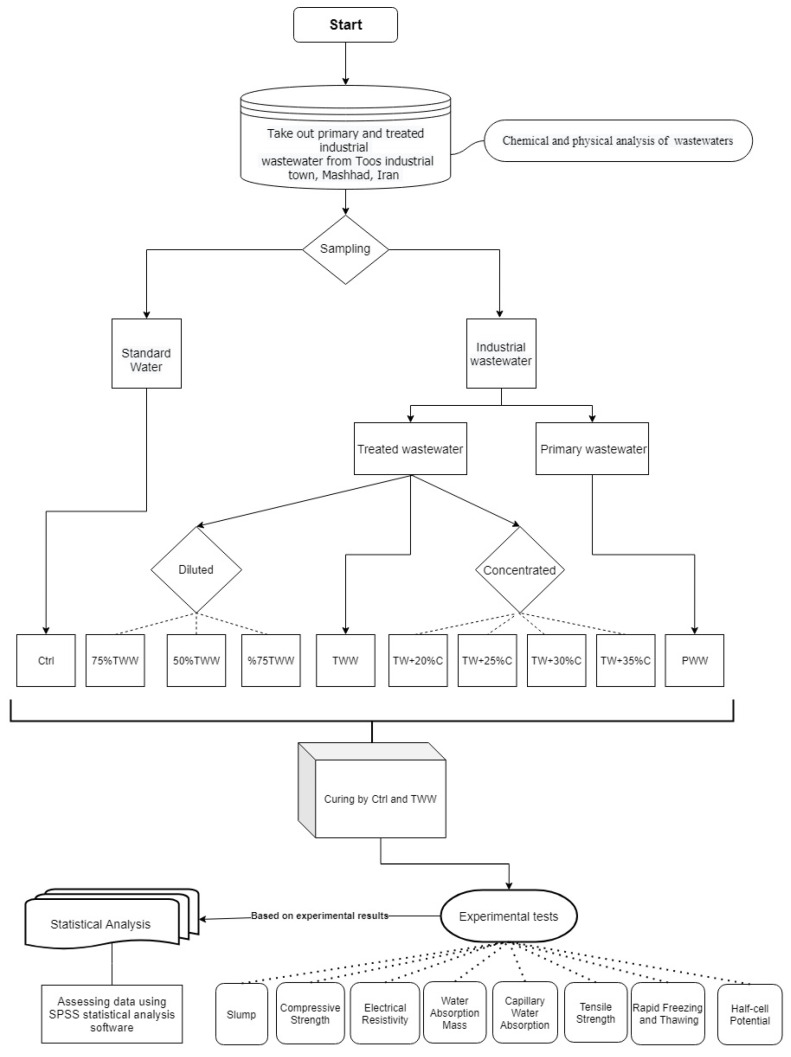
Flowchart of methodology.

The methodology consisted of several stages of operation and processing. Figure 1 represents the methodology strategy and functional stages in detail. Sampling, conditional stages, and experimental tests are the foundation of the methodology described in Figure 1.

In this research, ten different groups of specimens were produced with different wastewater concentrations. All groups of specimens had the same mix design, and no additive was used in order to figure out the exact effect of wastewater concentration on concrete durability and strength features. In this study, one of the targets was to find the optimum concentration of treated wastewater that may cause a less than 10% compressive strength loss compared with the control sample (made with drinkable standard water). Technically, 10% compressive strength reduction could still be counted as an acceptable substitution for the water in a concrete mix design [39]. The main control sample was produced with the potable water of Mashhad city. The used industrial wastewaters were categorized into four groups including treated wastewater, diluted treated wastewater, concentrated treated wastewater, and primary wastewater. All groups of concrete specimens were produced in a similar situation and were cured in drinkable water or treated wastewater according to the test standards and intended purposes. The parameters, such as concrete density, temperature, moisture, cement type and aggregates characteristics, were used in the same condition for all specimens.

### 2.2. Wastewaters

For producing concrete with wastewaters, the amount of distilled water was considered, based on the quality of the control specimen, and all other extra substances were subtracted. The majority of the time, there is an allowable limit for the water of mix design; within those restrictions, the impurity can be harmless and acceptable. Nevertheless, there is no limitation for organic materials in concrete and it is assumed that only wastewater impurities are the reasons for negative effects on the water in concrete mix design.

#### 2.2.1. Treated Wastewater (TWW)

Treated wastewater is also known as output wastewater and goes through three steps of refinement including filters, aeration and chlorination. Treated wastewater was used as the main replacement for drinkable water and for producing distilled and concentrated specimens as well. TWW was used for curing the specimens if they were intended to be cured by wastewater separately. The characteristics of TWW are presented in Table 2.

#### 2.2.2. Diluted Treated Wastewater (%TW)

Diluted specimens were produced by TWW plus mixing with distilled water. They contained 75% wastewater (75%TW), 50% wastewater (50%TW), and 25% wastewater (25%TW), respectively, and the remainder was distilled water. These water percentages of the mix designs were selected in order to investigate the existence of linear or non-linear relationships in strength and durability features by diluting treated wastewater as the water of the mix design. Based on the laboratory results, the number of parameters was reduced correctly by dilution percentages. In order to obtain the number of parameters in diluted specimens, the characteristics of treated wastewater (Table 2) should be reduced by dilution percentages.

#### 2.2.3. Concentrated Treated Wastewater (TW + %C)

Concentrated specimens were produced from TWW by evaporation; concentrating percentages were 20% (TW + 20%C), 25% (TW + 25%C), 30% (TW + 30%C), and 35% (TW + 35%C), respectively. Based on laboratory results, the parameters of the thickened specimens were increased by the concentration percentages. So, concentrated specimens had the same parameters as the treated wastewater (Table 2) but their characteristics were 20%, 25%, 30% and 35% more than the characteristics of the treated wastewater, respectively. According to the intended concentration, the amount of surplus treated wastewater was added and after time at a precise warming temperature, the intended concentration was achieved. Reaching the intended concentration by way of evaporation is almost acceptable, but sufficient accuracy for important parameters such as COD, BOD, Sulfate, Chromium, Cadmium and Salt was considered and double-checked.

#### 2.2.4. Primary Wastewater

The initial discharge of industrial wastewater is primary wastewater, which is from a collection of several polluting industries such as pharmaceutical, food, ironmaking and chemical. It contains many organic materials and caustic heavy metals such as Cadmium and Chromium because it does not go through any refinement process and, technically, this is the TWW before the refinement procedure. PWW contains a huge amount of organic materials, microorganisms and heavy metals, which are mostly harmful and caustic for both the environment and concrete. Table 2 shows the characteristics of primary wastewater (PWW). In Table 2, TDS, EC, COD, BOD and TSS stand for Total Dissolved Solids, Electrical Conductivity, Chemical Oxygen Demand, Biochemical Oxygen Demand and Total Suspended Solids, respectively.

### 2.3. Concrete Preparation

For producing the control sample and curing all groups with normal water, the potable water of Mashhad, Iran, was used. In order to conduct this research, concrete cubic samples (100 × 100 × 100 mm), including 400 kg cement per cubic meter, were made and tested; for each test, the required standard, specification and introduction were fully considered. The Portland cement type ΙΙ, produced by Mashhad cement factory, Mashhad (Iran), was chosen and its quality was tested according to the ASTM-C150. The strength class of the Portland cement was 42.5R, and the desired strength of the concrete was 35 Mpa, which was achieved based on breaking cylinder specimens and regarding the average result. Moreover, the concrete grade was M35, which was commensurate with the achieved results. The desired workability based on NS 8500 EN 165 was S3. The quality of the required material in the production of concrete, the chemical and physical analysis of water and wastewaters, and the sieve analysis of the aggregates were experimentally assessed. The resistance of degradation in large size coarse aggregate to abrasion and impact was carried out using a Los Angeles machine. In addition, the soundness of the aggregates was evaluated using the sodium sulfate or magnesium sulfate and sulfate content of the aggregates. In this research, the following parameters were also checked: the hydraulic cement autoclave expansion, the amount of water essential for an ordinary consistency of hydraulic cement, and the setting time of hydraulic cement using a Vicat needle. The ASTM C33 standard was adopted for checking the coarse and sand sizes using a sieve assessment and the precise percentage passing through sieve number 200 was determined. Table 3 shows the chemical and physical properties of the cement. Tor educe the effect of other parameters on the concrete, except for wastewater, a good-quality, continuous, less flawed aggregate was used [40]. The ASTM-C33 [27] standard was adopted to test the characteristics of the aggregates. It should be noted that the aggregates used in this study were kept in SSD condition. The mix design for all groups of specimens was the same and the concrete mixture is presented in Table 4. The concrete specimens were molded in metal molds and cured based on ASTM-C31 [41]. A separate set of specimens was cured by potable water, and a separate set of specimens was cured by treated wastewater. In addition, all requirements were considered in terms of curing and the storage of test specimens before rupture based on ASTM-C31 [41]. The concrete mixture is presented in Table 4. For curing purposes, the temperature in the laboratory was 25 centigrade while the relative humidity varied from %30 to %45 throughout the time of curing and testing. For both curing methods, curing in water and wastewater was conducted at the same time. All specimens were dried by oven prior to testing for water absorption.

To reach the optimum mix design, the ACI method of concrete mix design was used based on the water–cement ratio of 0.42 and, for the mechanical mixing of the cement, ASTM-C305 [42] was adopted. The good-quality and washed aggregates were selected after several initial samples according to the details in Table 5. In Appendix A, Table A1 and Table A2 present other details of the aggregates used in this study for constructing concrete.

## 3. Results and Discussion

### 3.1. Slump

**Figure 2:**
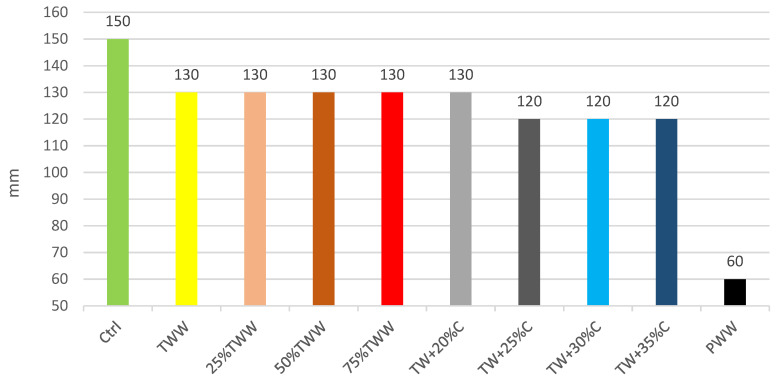
Slump test results.

The slump shall be consistent with the placement and consolidation methods, equipment, and site conditions and shall be identified by the contractor and concrete supplier prior to construction. According to the achieved results, TWW had less workability than the control sample. Diluted specimens reacted like TWW, which shows that the existence of the wastewater can affect the workability even at low percentages. The concentrated specimens followed the same method of treated TWW, but TW + 25%C had a reduction and stayed in the next specimens too. The TWW had 13.3% lower workability than the control specimen and by increasing the concentration of treated wastewater TW + 25%C by 25%, the workability declined 20% more than the control. It clearly showed that wastewater has a subtractive effect on workability and it is dependent on wastewater concentration. So, it is highly recommended, for projects with a high required workability, that the additives should be considered to increase the slump, especially when more concentrated wastewaters are used as the water of the mix design. No linear relationship was observed in any specimen when their concentration was increased or decreased. The concentrated specimens were more viscous and greasy, which is one of the reasons why concentrated specimens had less workability; it was obvious in the PWW sample, which had the highest impurities. Figure 2 represents the slump test results.

### 3.2. Compressive Strength

**Figure 3:**
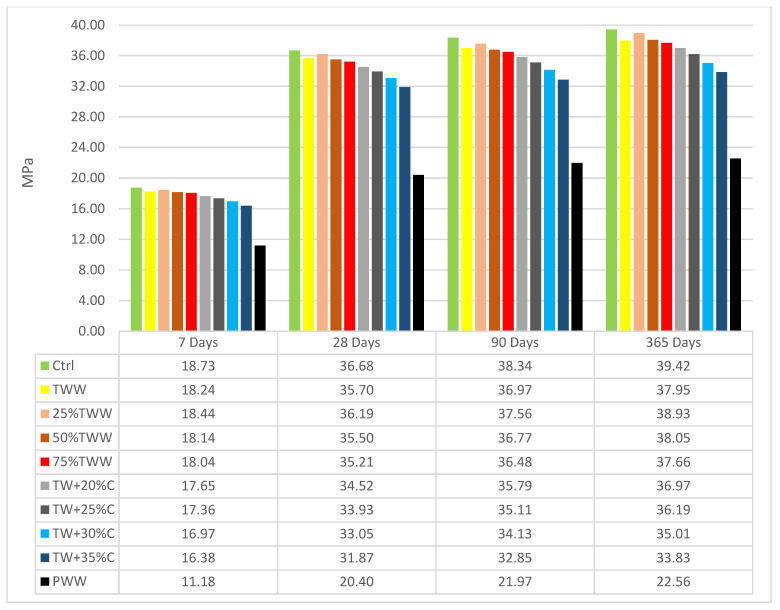
Compressive strength cured by drinkable water.

**Figure 4:**
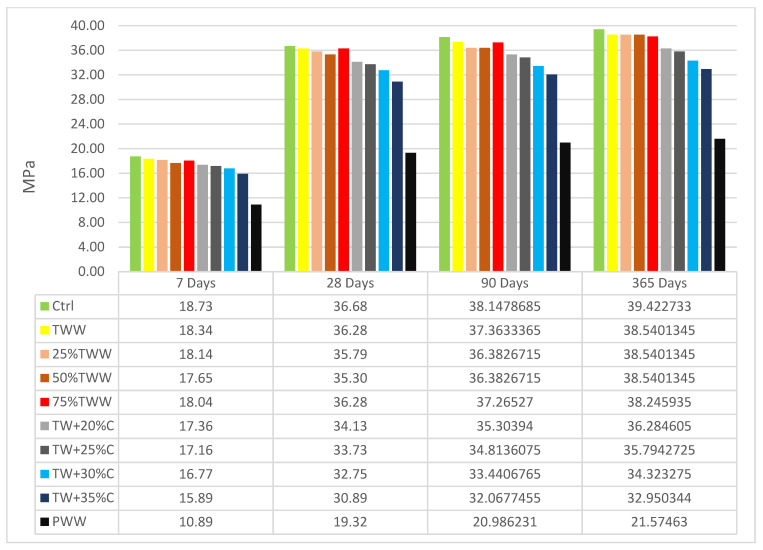
Compressive strength cured by treated wastewater.

The compressive strength results at different ages and days are shown for specimens cured by drinkable water (Figure 3) and cured by treated wastewater (Figure 4). The compressive strength was obtained by testing cubic specimens according to the BS EN 12390-3. The cubes were tested in a 3000 kN testing machine at a rate of 2.5 kN/s. The control sample had the highest strength at all ages, substantiating that the best result can be achieved by using drinkable water. TWW had lower strength than the control, but its reduction was insignificant. So, it demonstrated that treated industrial wastewater is applicable for use in concrete. The compressive strength in wastewater specimens was better when the concentrations of water for producing and curing were the same. It vividly showed the homogeneity and similarity features between the curing situation and water of the mix design. For instance, at the age of 7, 28, 90 and 365 days, when TWW and 75%TW were cured by treated wastewater, they had 0.54%, 1.65% 1.06%, 1.55%, 2.86%, 0%, 3.6% and 1.06% more compressive strength than when cured by standard water, respectively. Besides, 25%TW, in which its mixed design water was roughly similar to drinkable water, had 1.62%, 1.1%, 1.6% and 1.02% more compressive strength when it was cured by standard water at different ages. The concentrated specimens at low ages had a better performance when they were cured by treated wastewater but at late ages, better results were shown for those cured by drinkable water. The positive effect of curing with treated wastewater for those specimens produced by treated wastewater disappeared by increasing the specimens’ concentration and was changed adversely. For instance, TW + 35%C cured by treated wastewater had 3%, 3.1%, 2.4% and 2.6% less compressive strength when it was cured by treated wastewater than when it was cured by drinkable water. PWW produced by primary wastewater had the highest impurity, and corroborated this result, having 2.6%, 5.3%, 4.5% and 4.3% less compressive strength when it was cured by treated wastewater. Neither in the diluted specimens nor the concentrated specimens was a linear relationship was observed and a non-linear relationship was dominant; however, the concentration of specimens was increased and decreased in order.

By aging, concentrated specimens had less compressive strength growth than the control sample and, by increasing the specimens’ concentration, the reduction increased. One of the most important intentions of this study was to find the impurity and concentration of wastewater which causes a 10% reduction in the compressive strength of concrete in comparison to the control after 28 days. Based on Figure 3 and Figure 4, TW + 30%C, cured by drinkable water and treated wastewater at the age of 28 days, had 9.9% and 10.7% less compressive strength than the control, respectively. This clarified the largest amount of impurity in industrial treated wastewater which can be still acceptable for use in concrete mix design [36]. The chemical and physical characteristics of TW + 30%C were as follows: BOD: 150 mg/L, COD: 200 mg/L; Total Dissolved Solid: 1924 mg/L; Total Suspended Solids: 33 mg/L; Sulfate: 98 mg/L. It exhibited the optimum concentration of wastewater for refining to balance mechanical, durability and physical characteristics.

### 3.3. Electrical Resistivity

**Figure 5:**
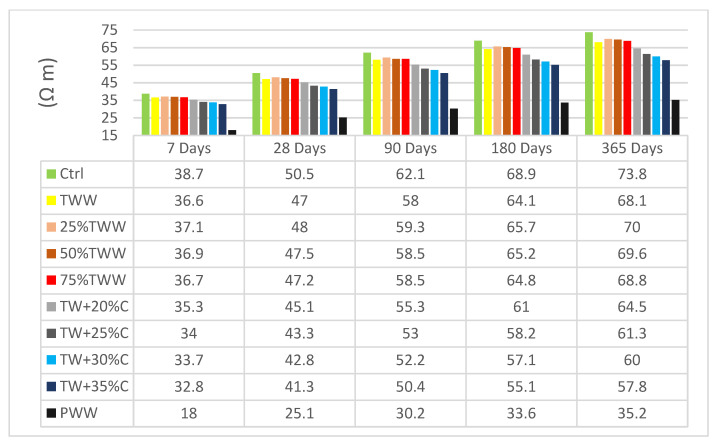
The results of concrete electrical resistivity tests cured by standard water.

**Figure 6:**
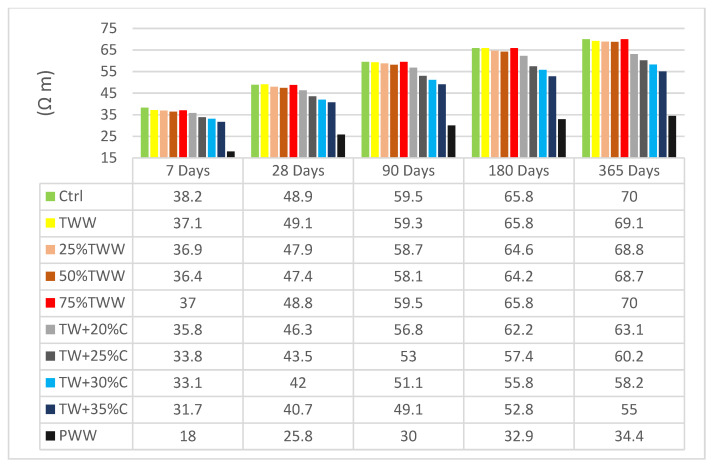
The results of concrete electrical resistivity tests cured by TWW.

The level of permeability of concrete has a direct effect on the electrical resistivity of specimens. This test indicates specimens’ permeability and specifies existing voids and cracks in the concrete structure, which have a significant effect on concrete durability [43]. Figure 5 and Figure 6 present the electrical resistivity of specimens at the age of 7, 28, 90, 180 and 365 days.

The control sample had better resistance when it was cured by drinkable water and a reduction was observed when cured by treated wastewater. By increasing the concentration, electrical homogeneity decreased. The diluted specimens’ behavior was inclined towards the TWW results and not the control, even when insignificant amounts of treated wastewater were involved. For example, 25%TW, which is 75% drinkable water, followed the TWW’s resistance and not that of the control, cured by either drinkable water or treated wastewater. It showed that whenever wastewater parameters are involved in the specimens, they could exceedingly influence the concrete’s structure and create voids and porosity in specimens. So, diluting the concentration has an insignificant effect on electrical resistivity enhancement. TWW and specimens with concentrations close to that of TWW had better resistivity when they were cured by treated wastewater at lower ages; however, with aging the positive effect declined even on them, as if being cured by treated wastewater in the long term has caustic effects on concrete structure and causes more avenues of penetration. Nevertheless, in specimens with a lower concentration at an early age, a resistance growth was observed which again supported the positive effect of homogeneity features, as well as the negative effect of being cured by treated wastewater in the long term.

At the age of 28 days, TW + 30%C, cured by drinkable and treated wastewater, had 15% and 14% less electrical resistivity than the control sample, respectively. This indicated that using wastewater in concrete has more negative effects on the concrete’s durability features than on its strength, because it had 10% reductions in the compressive strength test but a 15% reduction in electrical resistivity. Therefore, it is recommended that treated wastewater should not be used for projects with high contact with caustic material or marine projects.

### 3.4. Water Absorption Mass

For this test, specimens are dried using an industrial oven. In the drying process, heating and cooling at a special set of temperatures in both the oven and desiccator over a determined period are used. At every step, the materials are weighed. The workflow of a water absorption test is based on drying specimens in a laboratory or industrial oven according to BS1881-122 [30]. The time and temperature for using the oven and the cooling time are set for the test. Specimens are weighed both before and after the cooling. The test is followed by immersing the materials in the water, at 23 °C, for one day. Materials can be kept in the water either for the whole day or until equilibrium. Table 6 indicates the results of mass water absorption, which has a significant relationship with concrete permeability. The less porous the structure of concrete is and the less cracks it has, the less possibility exists for the movement of harmful parameters into the structure of the concrete; consequently, concrete corrosion is less expected. Hence, based on BS1881-122, the allowable water absorption is restricted to between 2% and 5%. In this test, except for PWW, all specimens stood in the allowable limitation after 72 h; however, TW + 35%C stood at the edge of rejection. This test showed not only that using wastewater increases water absorption, but also that the rate of age to age water absorption growth is more than that of the control, which is improper. For instance, control samples from 1 h to 72 h had 49.5% water absorption growth but TWW and TW + 30%C had 53.3% and 63.4%, respectively. The TW + 30%C had 39.5% more mass water absorption than the control, which shows the exactness of 30 min water absorption results. Table 6 shows the mass water absorption.

### 3.5. Capillary Water Absorption

The capillary test evaluates the process of non-saturated concrete water absorption by capillary suction while it is in touch with water. Table 7 shows the results of capillary water absorption at 3, 6, 24 and 72 h. Basically, the more moisture concrete contains, the less capillary water absorption will be measured. The capillary water absorption increased by using wastewater; even 25%TW, which contained 75 percent distilled water, had 11.19% more capillary water absorption than the control at 72 h. It showed that treated wastewater, even at low concentrations, influences capillarity absorption and subsequently reduces concrete durability. Using wastewater causes bigger and looser capillary pipes, which are connected to each other and intensify the concrete corrosion. The more and larger capillary pores a concrete has, the more deleterious substances will pass into it superficial and interior layers. For instance, after 72 h, TWW samples had 25.87% more capillary water absorption growth than the control; this growth for TW + 30%C was 95.30%.

**Figure 7:**
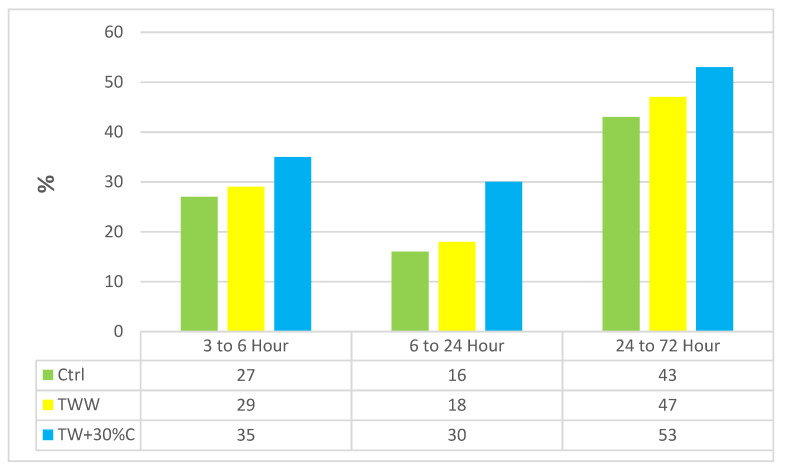
The capillary water absorption growth hour to hour (%).

Figure 7 indicates the rate of growth during the test period. The wastewater specimens had more capillary water absorption and growth rates than the control sample. For example, from 24 to 72 h, TWW and TW + 30%C had 4% and 10% more growth than the control. So, based on Table 8, Table 9 and Table 10, it is highly recommended that wastewater at high concentrations should not be used as the water of concrete mix design when it is going to be used in caustic environments because of the high possibility of corrosion.

### 3.6. Tensile Strength

**Figure 8:**
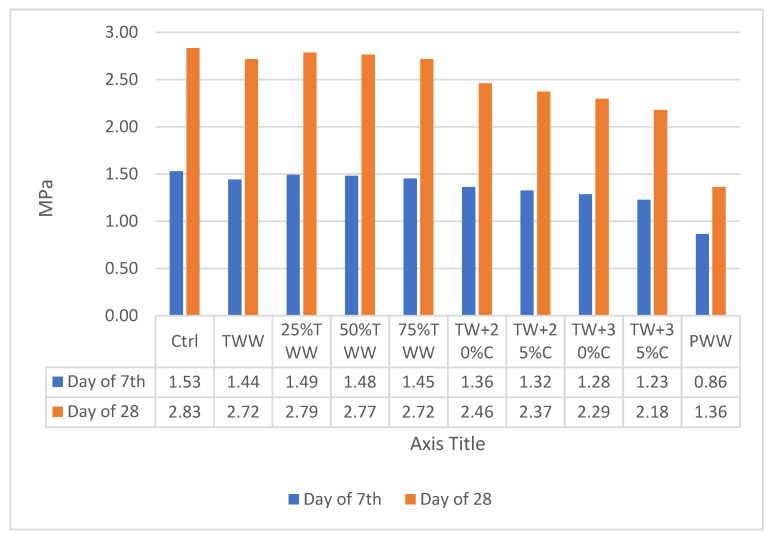
The results of tensile strength cured by drinkable water.

**Figure 9:**
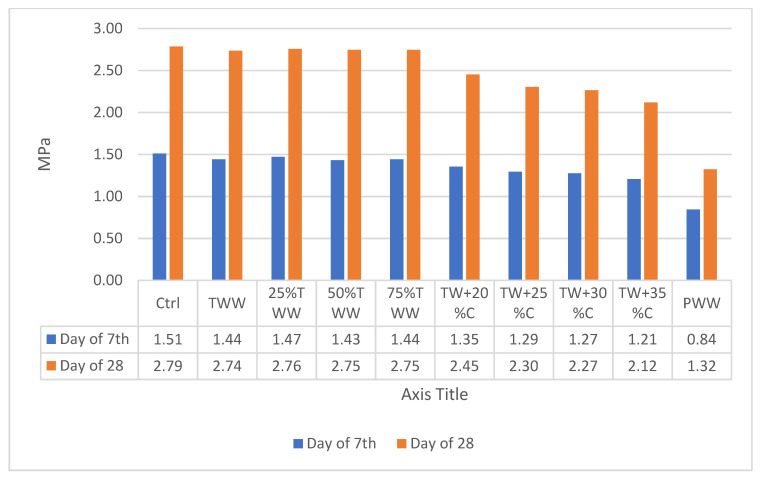
The results of tensile strength cured by treated wastewater.

The tensile strength of concrete is a prominent property when it is to be utilized for making prestressed concrete structures, roads and runways. Figure 8 and Figure 9 illustrate the results of tensile strength testing on days 7 and 28 on concrete cured by drinkable water and treated wastewater, respectively. This test illustrated that the behavior of specimens in the tensile strength test is approximately similar to that in the compressive strength test but the situation is worse in concentrated specimens. For example, the TW + 30%C sample cured by drinkable water and treated wastewater on day 28 had almost 10% less compressive strength than the control and it had 19% less tensile strength. This indicated that Interfacial Transition Zone (ITZ) area is weaker in wastewater specimens and the capacity for water absorption is more in this area. Some wastewater parameters, such as sludge, have spongy features and they reduce the water available for hydration reactions, while the water–cement ratio needs to be more in the ITZ region [39]. In addition, some other greasy wastewater, such as oils, cover the aggregates’ surface and hamper the proper connection between cement and aggregates in the ITZ region [39]. That is why the tensile strength is more affected by increasing the concentration compared to the compressive strength. Although the specimens’ concentration was increased and decreased in order, no linear relationship was observed between diluted or concentrated specimens.

Not only did the tensile strength decline by increasing the concentration, but the rate of tensile strength growth was also lower than in the control sample. For instance, within days 7 to 28, the control sample cured by drinkable water and treated wastewater had 85.3% and 84.4% growth, but TW + 30%C had 78% and 77% tensile strength growth and TW + 35%C had 77% and 75% growth, respectively.

### 3.7. Rapid Freezing and Thawing

Table 8 indicates the compressive strength results of specimens at the age of 28 days, and the strength reduction of each cycle due to the rapid freezing and thawing test. Change in compressive strength: a decline of more than 10% is the sign of failure. The volume expansion is the first reason for cracking in concrete. This expansion is caused by frozen water inside the concrete. Another reason for cracking is thermal stress. Thermal stresses appear because of repeated freeze–thaw cycles. By increasing the number of fast freeze–thaw cycles, the value of the mechanical property declines. Basically, existing air bubbles in the concrete are effective at enhancing its resistance to disintegration when exposed to cycles of freezing and thawing in a censoriously saturated state, and at decreasing the scaling that involves the application of chemicals for ice removal [44]. The tiny air voids work as empty chambers in the paste for the freezing and moving water to enter, hence mitigating the pressure in the pores and intercepting damage to the concrete. However, it is very unlikely that air-void clustering can happen, causing a loss of compressive strength. Likewise, the pore connectivity, as well as pore-size distribution, are known as the main factors which remarkably affect the freezing and thawing resistance [45].

The recommended bubble distribution is less than 0.2 mm, but by using wastewater instead of drinkable water, the pores become harmful for the concrete [46]. The better pore connectivity leads to a better performance of the concrete regarding the freezing and thawing test, so the higher the connectivity in the concrete, the higher the resistance of the specimen [47]. The impurities of wastewaters disrupt the formation of appropriate air voids, pore structure and pore-size distribution in the concrete. Consequently, low connectivity of pores and larger harmful pores (pores greater than 0.064 μm) are developed. By using wastewater with high concentrations, the free spacing and pore structure are not formed properly and somehow get clogged by TDS and other wastewater impurities, including lead, cadmium and detergent [48]. Therefore, those tiny air voids do not act like chambers in wastewater specimens in comparison with the control sample produced by standard drinkable water. Technically, the air bubbles in the concrete provide protection from the strain that originated from the freezing of water in the capillary gaps in the concrete specimens and thus minimize damage to the hardened paste.

Results indicated that the reduction rate in the control and TWW samples was almost the same until 100 cycles, but then TWW demonstrated different behavior and declined more than the control sample. For instance, until 100 cycles, in comparison to day 28 compressive strength, the control and TWW had an almost 3% strength reduction, whereas in 150 and 200 cycles, the control sample had 4% and 5% reductions while TWW had 6% and 7.6% compressive strength reduction, respectively. It was observed that the strength of the specimens was negatively affected by the lack of proper pore connectivity and air voids in the specimens produced by wastewater, especially when the smooth exterior layer of the specimens was gone due to initial cycles of the test, and the inner pore structures were the main effective factor. This illustrated the destructive effect of harmful pores and tiny air voids on the concrete structure caused by wastewater impurities. Interestingly, 75%TW also had the same reaction as TWW and the rate of reduction rose after cycle 100. No significant difference was observed in 25%TW; its concentration was fairly close to that of the control specimen and it supported the negative impact of larger pores in the concrete structure even after using diluted wastewater. Compared to the control sample, concentrated specimens had more compressive strength reduction in all cycles, and by increasing the concentration and cycles, the rate of reduction increased. Clearly, PWW, which contained the highest impurity, had the lowest strength due to having harmful pores. It should be noted that the exterior layer of PWW was already honeycombing with vulnerability, yet cracks and flaws intensified the volume expansion and failure in this specimen.

**Figure 10:**
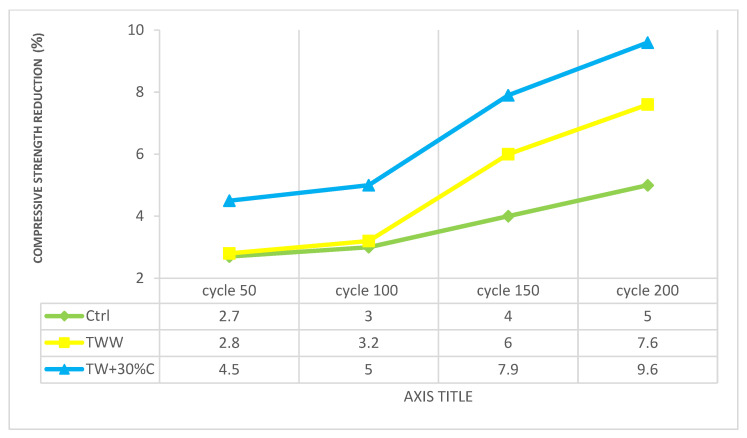
The compressive strength reduction in each rapid freezing and thawing cycle.

Figure 10 shows the compressive strength reduction rate due to rapid freezing and thawing, which has a direct relationship with poor void parameters and porosity connectivity in the concrete structure. In fact, by having more sulfate, TDS, BOD and COD in the utilized wastewaters, the availability of well-developed voids decreased, which increased the pressure in the concrete. The existing oil and impurities in the wastewater led to losing the connectivity of the pores, and then freezing and thawing resistance declined. Again, these reduction rate differences were more conspicuous after 100 cycles when the pressure reached a peak. For example, by concentrating the TWW up to 35%, the comprehensive strength after 200 cycles of compression with the control and TWW dropped by 26% and 19%, respectively. 

Using wastewater causes larger pores and more voids in the concrete’s structure and these specimens can contain water and, subsequently, more frozen water inside of the concrete and more corrosion are expected in the rapid freezing and thawing test. TW + 30%C at 50, 100, 150 and 200 cycles had 12%, 13.9%, 17.4% and 21.4% less compressive strength than the control. The rate of compressive strength reduction was higher in the specimens with higher concentrations. Therefore, the improper pore structures and clogged air voids caused by the total dissolved and all impurities in the more concentrated wastewaters can be interpreted as the primary factors of strength reduction in the concrete specimens produced by wastewater. Based on the achieved results, void parameters, air voids and size-distribution have more prominent effects on the resistance than the air-void spacing. Therefore, based on the obtained results, air void structure in air-entrained concretes utilizing a Protected Paste Volume (PPV) parameter is recommended as it protects the paste area with air voids in the total paste area.

### 3.8. Half-Cell Potential

Table 9 illustrates the half-cell potential, which is influenced by chloride ions and the internal alkaline environment of concrete. Basically, increasing the wastewater concentration caused a larger reduction in the reinforcement corrosion potential. In other words, by aging the specimens and increasing the concentration of used wastewater as the water of the mixture design, the possibility of corrosion increased [39]. For instance, the corrosion in TW + 30%C and PWW started after 24 and 8 days; this is clearly because of the wastewater parameters. Half-cell potential decreased when chloride content and sulfate content increased in the wastewaters’ concentration. In fact, those specimens with higher compressive strength demonstrated better half-cell potential. Likewise, the extent of corrosion escalated with the reduction of the half-cell potential. According to the voltage, corrosion was conspicuous when the half-cell potential was lower than −450 mV in dry conditions. In addition, using more concentrated wastewater not only decreased the level of half-cell potential but it also negatively affected the level of corrosion and the age at which corrosion starts. For instance, in PWW, which had the highest chloride and sulfate in comparison to other specimens, corrosion occurred almost four times sooner than in the control or even in TWW. Clearly, the level of corrosion sped up with the decline of the half-cell potential.

### 3.9. Statistical Analysis

In scientific research, researchers seek to present results as practically and, of course, as easily as possible. One of the most important ways that other researchers can make practical use of research is to provide mathematical models for use in future experiments and research [49]. In this study, after completing the laboratory phase, the authors collected laboratory data to examine the data’s relationships. Data were analyzed using SPSS statistical analysis software. In this statistical analysis, some input parameters were used, including TDS, TSS, EC, Detergent, Sulfate, Chromium and Cadmium, to predict output parameters including Compressive Strength, Electrical Resistivity, Tensile Strength, Rapid Freezing and Thawing, Water Absorption in 30 min, Water Absorption Mass 72 h, and Capillary Water Absorption 72 h. The statistical indicators of all parameters are shown in Table 10. Standard division was reported using Equation (1).

(1) $$\sigma =\sqrt{\frac{\sum {\left(x_{i}-\mu \right)}^{2}}{N}}$$

where *σ*, *N*, *x_i_* and *µ* are the population standard deviation, the size of the population, each value from the population and the population mean, respectively.

In the next step, the normality of the data should be checked. The normality of data is generally determined by examining the Skewness and Kurtosis coefficients [50,51,52]. Achieving a situation where data distribution is perfectly normal is very rare, so in scientific texts, data is normal when the coefficients of Kurtosis and Skewness are in the range of −2 to 2 [53]. According to the coefficients of Kurtosis and Skewness in Table 11, all variables have a normal distribution. Once the normality of the data is determined, it is time to determine the correlation coefficient between the variables. For normal data, the Pearson correlation test is used. Table 11 presents the results of the Pearson correlation test.

Once the correlation coefficients have been determined, it is time to determine the estimation models for research outputs. Multivariate linear regression is used for this purpose. The general form of multivariate linear regression is as follows [54,55,56]:

Y = a_0_ + a_1_X_1_ + a_2_X_2_ + a_3_X_3_ +…, (2)

where Y is the dependent variable (output of model), X_i_ is the independent variable (inputs of the model), and ai are the regression coefficients of the model. The models obtained from the analysis performed with SPSS software are presented in Table 12. To measure the accuracy of the models, the two criteria R^2^, standard error and standard deviation were used.

The equations presented in Table 12 are very accurate, but they include an important point. These equations were constructed based on the laboratory data of this study. If the number of laboratory data is increased, the coefficients of the models will change slightly. Therefore, the authors recommend that other researchers, before applying these equations, first calibrate the models for their project or research conditions and then use them.

In sum, the achieved results were commensurate with other tests with data integrity, and no significant contradiction was observed. The negative impact of the wastewater was conspicuous in durability results and curing with wastewater is not recommended. This research indicated that using industrial wastewater could decrease the quality of produced concrete according to its concentration but, based on the results, a proper understanding of the use of different concentrations was presented, which helps to reach the economic level of refinement in industrial towns for using their wastewater in concrete.

## 4. Conclusions

In this paper, ten groups of concrete specimens with different industrial wastewater concentrations were produced and cured by drinkable water and treated wastewater separately according to tests and standards. Using wastewater as the water for mix design reduces the strength and durability, but industrial TWW can be a good and acceptable replacement for the water in mix design, having insignificant strength and durability reduction effects on concrete in all tests. By concentrating the treated wastewater properties by up to 30% in the TW + 30%C specimen, the compressive strength declined by almost 10% after 28 days, which showed the optimum level of concentration for an industrial town’s refinement. However, concentration had more adverse effects on durability tests, which showed that using wastewater causes more negative impacts on the durability than on the strength features of concrete. PWW did not have acceptable behavior in any tests, and it was rejected. Although in concentrated and diluted specimens the percentage of wastewater was increased or decreased in order, no linear relationship in the strength and durability features of the tests was observed. Control specimens showed better strength and durability when they were cured by drinkable water, which proved homogeneity and similarity features; nonetheless, wastewater specimens showed better strength and durability when they were cured by wastewater only at lower ages, whereas the good effect disappeared at late ages; as if being in touch with treated wastewater for curing deteriorates the specimens’ strength and causes corrosion. All of the specimens had less growth in terms of strength and durability when they were cured by treated wastewater in comparison to being cured by drinkable water. Using wastewater reduced the electrical resistivity and increased the water absorption of samples, yet diluting the treated wastewater could not correct the negative effects on concrete durability. Diluted specimens’ results were closer to those of TWW, not of the control and, by increasing the concentration, negative effects became more conspicuous. Using wastewater in the concrete had a negative impact on the rapid freezing and thawing results. It is concluded that the improper pore-size distribution, lack of pore connectivity and air voids caused by total dissolved and impurities of the wastewaters can be counted as the main reasons of strength reduction in the specimens produced by wastewater. Despite dilution, it was not an adequate solution for resolving the strength reduction of specimens in the freezing and thawing test, and even low concentrations of wastewater disrupt the formation of appropriate air voids and pore structure void parameters in the wastewater specimens in comparison with the control. By concentrating industrial wastewater, not only were the freezing–thawing properties negatively affected, but also the strength reduction rate increased, especially after 100 cycles. In the half-cell potential test, using wastewater insignificantly damaged the reinforcement, but specimens with more concentrated wastewater started to corrode faster. The specimens’ appearances had insignificant differences, except for PWW, which had more discontinuity and a distinguishable lack of hydration; however, by increasing the concentration, uneven and small cracks on the exterior layers were observed. Using wastewater increased water absorption and decreased workability. Therefore, it is highly recommended that it not be used in projects with caustic materials, exposed surfaces, or when a high slump is needed.

## Figures and Tables

**Table 1:** The types of testing and the corresponding Standards.

Type of Testing	Method of Testing
Chemical and physical properties of treated wastewater	APHA [22]
Standard Specification for Mixing Water Used in the Production of Hydraulic Cement Concrete	ASTM C1602M-18 [23]
Standard specification for Portland cement	ASTM-C150 [24]
Standard test method for density of hydraulic cement	ASTM C188-15 [25]
Standard test method for sieve analysis of fine and coarse aggregates	ASTM-C136 [26]
Standard specifications of concrete aggregates	ASTM-C33 [27]
Standard test methods for the time of setting of hydraulic cement by Vicat needle	ASTM-C191 [28]
The slump of hydraulic-cement concrete	ASTM C143 [29]
Testing hardened concrete. Compressive strength of test specimens	BS EN 12390-3 [30]
Standard test method for splitting tensile strength of cylindrical concrete	ASTM-C496 [31]
Absorption of concrete water	BS1881-122 [32]
Florida method of test For Concrete resistivity as an electrical indicator of its permeability	FM-5-578 [33]
Water absorption rate by hydraulic-cement concretes	ASTM-C1585 [34]
Concrete resistance against thawing and rapid freezing	ASTM-666 [35]
The standard method for the test of half-cell potentials of uncoated reinforcing steel	ASTM- C876-15 [36]
Standard test method for density, absorption, and voids in hardened concrete	ASTM C642-13 [37]
Standard test method for air content of freshly mixed concrete by the pressure method	ASTM C231/M17a [38]

**Table 2:** Chemical and physical characteristics of treated wastewater and primary wastewater.

No.	Parameter	Unit	Treated Wastewater	Primary Wastewater	Mashhad Potable Water (Ctrl)
1	pH	-	7.92	7.68	7.2
2	TDS	mg/L	1870	2541	412
3	SALT	mg/L	2.4	2.51	40
4	EC	mg/L	3950	4120	193
5	COD	mg/L	150	3215	0
6	BOD	mg/L	114	1240	3
7	TSS	mg/L	25	451	121
8	Ammonium	mg/L	2	3	0.4
9	Detergent	mg/L	1.25	3.1	-
10	Color	-	Light brown	Black	Transparent
11	Temperature	°C	17	17–19	25
12	Sulfate	mg/L	80	145	50
13	Chloride	mg/L	1230	740	94
14	Chromium	mg/L	0.9	1.89	0.1
15	Cadmium	mg/L	0.7	2.95	-
16	Lead	mg/L	2.85	2.85	0.02
17	Turbidity	Nephelometric Turbidity Unit	10	800	2

**Table 3:** Chemical and physical properties of cement.

Chemical & Physical Measurands	Units	Test Method	ISIRI 389	EN 197-1: 2011	Sample Analysis
SiO_2_	%	ASTM C114:2011b	>20.00	-	21.77
Al_2_O_3_	%	ASTM C114:2011b	<6.00	-	5
Fe_2_O_3_	%	ASTM C114:2011b	<6.00	-	4.3
CaO	%	ASTM C114:2011b	-	-	63.13
mgO	%	ASTM C114:2011b	<5.00	<5.00	1.78
L.I.O	%	EN 196-2:2013	<3.00	<5.00	1.38
SO_3_	%	EN 196-2:2013	<3.00	<3.5	2.22
IR	%	EN 196-2:2013	-	<5.00	0.63
Na_2_O	%	EN 196-2:2013	-	-	0.32
K_2_O	%	EN 196-2:2013	-	-	0.83
CI	%	EN 196-2:2013	-	<0.10	0.010
Free CaO	%	EN 196-2:2013	-	-	1.10
Cao/SIO_2_	-		-	>2.0	2.90
C_3_S + C_2_S	%		-	>66.667	73.48
Fineness	cm^2^/gr		>2800	-	3000
Le Chatelier Expansion	mm	EN 196-3:2005	-	<10.00	0.9
Initial Setting Time	min	EN 196-3:2005	>45	>75	116
Final Setting Time	min	EN 196-3:2005	<360	-	175
3 days Com. Strength	MPa	EN 196-3:2005	-	-	16.8
7 days Com. Strength	MPa	EN 196-3:2005	-	-	23.2
28 days Com. Strength	MPa	EN 196-3:2005	-	>32.5, <52.5	45.3

**Table 4:** The detail of concrete mixture designs.

Sample	Free Water Mass	Wastewater Mass	Cement Mass	Sand Mass
Control (Ctrl)	168 kg	-	400 kg	974 kg
Treated wastewater (TWW)	-	168 kg	400 kg	974 kg
Concentrated treated wastewater (TW + %C)	-	168 kg	400 kg	974 kg
Diluted treated wastewater (%TW)		168 kg	400 kg	974 kg

**Table 5:** Detail of mix design of concrete samples.

Parameter	Control (Ctrl)	Treated Wastewater (TWW), Concentrated Treated Wastewater (TW + %C), Diluted Treated Wastewater (%TW)	Primary Wastewater (PWW)
Free water mass	168 kg	-	-
Wastewater mass	-	168 kg	168 kg
Cement mass	400 kg	400 kg	400 kg
Sand mass	974 kg	974 kg	974 kg
Fine gravel mass	185 kg	185 kg	185 kg
Coarse gravel mass	576 kg	576 kg	576 kg
Stone powder	74	74	74

**Table 6:** Mass water absorption.

	1 H (%)	3 H (%)	24 H (%)	72 H (%)
Ctrl	2.10	2.62	2.93	3.14
TWW	2.42	3.05	3.54	3.71
25%TW	2.30	2.78	3.05	3.2
50%TW	2.30	2.85	3.25	3.45
75%TW	2.34	2.94	3.38	3.52
TW + 20%C	2.55	3.21	3.60	3.88
TW + 25%C	2.64	3.39	3.85	4.18
TW + 30%C	2.68	3.48	4.00	4.38
TW + 35%C	2.90	3.83	4.40	4.92
PWW	8.6	11.04	12.45	13.60

**Table 7:** The results of capillary water absorption ($\frac{gr}{mm^{2}}$ or mm).

Sample	3 H (%)	6 H (%)	24 H (%)	72 H (%)
CTRL	1.35	1.72	2.00	2.86
TWW	1.66	2.14	2.54	3.70
25%TW	1.46	2.05	2.28	3.18
50%TW	1.53	2.12	2.40	3.38
75%TW	1.57	2.08	2.40	3.44
TW + 20%C	1.80	2.35	2.84	4.22
TW + 25%C	1.88	2.52	3.18	4.86
TW + 30%C	1.94	2.62	3.40	5.22
TW + 35%C	2.05	2.84	3.70	5.65
PWW	8.90	12.88	16.85	29.32

**Table 8:** The results of resistance of concrete to rapid freezing and thawing.

Samples	28-Day Compressive Strength (MPa)	50 Cycles (MPa)	100 Cycles (MPa)	150 Cycles (MPa)	200 Cycles (MPa)
Ctrl	36.68	35.70	34.62	33.24	31.58
TWW	35.70	34.13	33.05	31.09	28.73
25%tw	36.19	35.30	34.23	32.75	31.09
50%tw	35.50	34.42	33.34	31.97	29.62
75%tw	35.21	34.13	33.15	31.28	29.13
TW + 20%C	34.52	33.54	32.26	30.01	27.46
TW + 25%C	33.93	32.36	30.99	28.54	25.99
TW + 30%C	33.05	31.38	29.81	27.46	24.81
TW + 35%C	31.87	29.91	28.34	25.99	23.44
PWW	20.40	18.34	16.87	15.20	13.34

**Table 9:** The start age of armature corrosion.

Sample	Age of Corrosion Start (Day)
Ctrl	32
TWW	27
25%TW	30
50%TW	28
75%TW	28
TW + 20%C	24
TW + 25%C	24
TW + 30%C	24
TW + 35%C	22
PWW	8

**Table 10:** Statistical characteristics of variables.

Variables	Mean	Maximum	Minimum	Kurtosis	Skewness	Variance	Std. Deviation
TDS (mg/L)	1879.2500	2431.00	1014.00	−1.249	−0.522	272,887.933	522.38677
EC (mg/L)	3935.5000	3959.00	3880.00	−0.461	−1.251	1064.000	32.61901
TSS (mg/L)	25.6250	38.00	10.00	−1.607	−0.306	108.517	10.41713
Detergent (mg/L)	1.2587	1.80	0.70	−1.494	−0.206	0.152	0.39002
Sulfate (mg/L)	77.0000	101.00	36.00	−1.169	−0.680	596.267	24.41857
Chromium (mg/L)	0.8400	0.99	0.50	0.553	−1.242	0.026	0.16199
Cadmium (mg/L)	0.7463	0.90	0.50	0.386	−0.854	0.015	0.12099
Compressive Strength (kg/cm^2^)	351.2500	370.00	315.00	−0.221	−0.784	281.133	16.76703
Electrical Resistivity (Ω m)	45.4938	49.10	40.70	−1.340	−0.439	7.898	2.81033
Tensile Strength (kg/cm^2^)	25.7875	28.40	21.60	−1.625	−0.346	6.276	2.50516
Rapid Freezing and Thawing (kg/cm^2^)	350.3750	370.00	322.00	0.076	−0.842	245.411	15.66559
Water Absorption in 30 min (%)	1.9137	2.30	1.72	−0.829	0.826	0.050	0.22328
Water Absorption Mass 72 h (%)	3.9050	4.92	3.20	−0.031	0.708	0.317	0.56346
Capillary Water Absorption 72 h (%)	4.2063	5.65	3.18	−1.504	0.491	0.874	0.93496

**Table 11:** Pearson correlation coefficients between inputs and outputs in this study.

		Inputs						
		TDS	EC	TSS	Detergent	Sulfate	Chromium	Cadmium
Outputs	Compressive Strength	−0.793	−0.525	−0.834	−0.85	−0.747	−0.678	−0.831
	Electrical Resistivity	−0.81	−0.536	−0.858	−0.856	−0.77	−0.688	−0.817
	Tensile Strength	−0.898	−0.661	−0.933	−0.934	−0.868	−0.777	−0.874
	Rapid Freezing and Thawing	−0.853	−0.658	−0.879	−0.891	−0.819	−0.796	−0.889
	Water Absorption in 30 min	0.842	0.602	0.888	0.896	0.807	0.728	0.832
	Water Absorption Mass 72 Hour	0.896	0.697	0.927	0.942	0.869	0.831	0.902
	Capillary Water Absorption	0.906	0.673	0.942	0.942	0.876	0.807	0.886

**Table 12:** Prediction models for determining characteristics of concrete.

Equations	R^2^	Std. Error	Std. Deviation
Compressive Strength=2504.102−0.017TDS−4.693TSS−24.11Detergent+2.869Sulfate+93.896Chromium−134.054Cadmium−0.551EC	0.964	4.37321	61.84
Electrical Resistivity=467.794−1.426TSS+7.087Detergent+0.522Sulfate+15.7Chromium−17.614Cadmium−0.110EC	0.960	0.76933	10.88
Tensile Strength=280.918+0.001TDS−0.066EC−0.758TSS+2.536Detergent+0.197Sulfate+19.169Chromium−17.064Cadmium	0.994	0.25981	3.68
Rapid Freezing and Thawing=3634.478−0.021TDS−0.847EC−7.396TSS−1.118Detergent+4.532Sulfate−24.176Chromium−63.520Cadmium	0.999	0.0001	0.0015
Water Absorption in 30 min=0.0006092TDS+0.011EC+0.112TSS−0.19Detergent−0.051Sulfate−0.739Chromium+0.945Cadmium−39.212	0.998	0.0002	0.0029
Water Absorption Mass 72 hours=0.02EC+0.178TSS+1.037Detergent−0.104Sulfate+0.515Chromium+1.866Cadmium−74.792	0.997	0.0005	0.0071
Capillary Water Absorption 72 HOUR=0.001TDS+0.032EC+0.389TSS−1.269Detergent−0.161Sulfate−1.902Chromium+2.821Cadmium−117.984	0.999	0.0001	0.0015

## Data Availability

Not applicable.

## Appendix A

This Appendix presents the granular details of the materials used in this study for producing concrete.

**Table A1:** Gradation test results.

Sieve		Percentage Passing					
No.	Size (mm)	Coarse Gravel	Fine Grave	Sand	Fine Sand	Stone Powder	Final Composition
1⅟₂ inch	37.5	100	100	100	100	100	100
1 inch	25	100	100	100	100	100	100
3/4 inch	19	93	100	100	100	100	98
1/2 inch	12.5	28	99	100	100	100	78
3/8 inch	9.5	1	78	100	100	100	67
No. 4	4.75	0.2	3	92	98	100	55
No. 8	2.38	0.2	0.6	71	95	100	43
No. 16	1.19	0.1	0.5	42	91	100	27
No. 30	0.6	0.1	0.5	23	88	98	17
No. 50	0.3	0.1	0.5	9.3	69	87	9
No. 100	0.15	0.1	0.5	3.1	20	70	5
No. 200	0.075	0.1	0.5	1.7	16	57	3

**Table A2:** Aggregates’ parameters.

Parameter	Sand	Fine Gravel	Coarse Gravel	Stone Powder
Specific density (kg/m^3^)	1730	1582	1576	2600
SSD density (kg/L)	2.580	2.70	2.71	2.70
Water absorption (%)	2.49	0.910	0.670	0.00
Sand equivalent	82	-	-	-
Passed from #200 (%)	1.70	0.5	0.1	56.60
Fineness modulus	3.60	6.16	7.05	0.45
